# Prediction of breast cancer sensitivity to neoadjuvant chemotherapy based on status of DNA damage repair proteins

**DOI:** 10.1186/bcr2486

**Published:** 2010-03-05

**Authors:** Hideki Asakawa, Hirotaka Koizumi, Ayaka Koike, Makiko Takahashi, Wenwen Wu, Hirotaka Iwase, Mamoru Fukuda, Tomohiko Ohta

**Affiliations:** 1 Division of Breast and Endocrine Surgery, Department of Surgery, St. Marianna University School of Medicine, Kawasaki, 216-8511 Japan; 2 Department of Translational Oncology, St. Marianna University Graduate School of Medicine, Kawasaki, 216-8511 Japan; 3 Department of Diagnostic Pathology, St. Marianna University School of Medicine, Kawasaki, 216-8511 Japan; 4 Department of Breast and Endocrine Surgery, Kumamoto University, Honjo 1-1-1, Kumamoto 860-8556, Japan

## Abstract

**Introduction:**

Various agents used in breast cancer chemotherapy provoke DNA double-strand breaks (DSBs). DSB repair competence determines the sensitivity of cells to these agents whereby aberrations in the repair machinery leads to apoptosis. Proteins required for this pathway can be detected as nuclear foci at sites of DNA damage when the pathway is intact. Here we investigate whether focus formation of repair proteins can predict chemosensitivity of breast cancer.

**Methods:**

Core needle biopsy specimens were obtained from sixty cases of primary breast cancer before and 18-24 hours after the first cycle of neoadjuvant epirubicin plus cyclophosphamide (EC) treatment. Nuclear focus formation of DNA damage repair proteins was immunohistochemically analyzed and compared with tumor response to chemotherapy.

**Results:**

EC treatment induced nuclear foci of γH2AX, conjugated ubiquitin, and Rad51 in a substantial amount of cases. In contrast, BRCA1 foci were observed before treatment in the majority of the cases and only decreased after EC in thirteen cases. The presence of BRCA1-, γH2AX-, or Rad51-foci before treatment or the presence of Rad51-foci after treatment was inversely correlated with tumor response to chemotherapy. DNA damage response (DDR) competence was further evaluated by considering all four repair indicators together. A high DDR score significantly correlated with low tumor response to EC and EC + docetaxel whereas other clinicopathological factors analyzed did not.

**Conclusions:**

High performing DDR focus formation resulted in tumor resistance to DNA damage-inducing chemotherapy. Our results suggested an importance of evaluation of DDR competence to predict breast cancer chemosensitivity, and merits further studying into its usefulness in exclusion of non-responder patients.

## Introduction

Recent advances in chemotherapy have significantly improved the prognosis of breast cancer patients. However, prediction of tumor sensitivity to chemotherapy has not reached a high level of confidence, whereas determining sensitivity to hormone therapy or trastuzumab is relatively more established. Estrogen receptor (ER), progesterone receptor (PR) and human epidermal growth factor receptor (HER)2/ErbB2 are practical benchmarks to exclude non-responding patients, and tailoring treatment based on gene status significantly optimizes the response rate of hormone therapy and trastuzumab, respectively. Prediction of chemosensitivity with equivalent accuracy is currently anticipated to further improve breast cancer prognosis.

Anthracycline-based regimens, such as epirubicin plus cyclophosphamide (EC), and taxanes represent the major chemotherapeutic agents used in the breast cancer field [1,2]. Of these, anthracycline-based chemotherapy induces DNA double-strand breaks (DSBs) [3,4], the most cytotoxic DNA lesion, that leads cells into apoptosis especially when relevant repair pathways (represented by homologous recombination (HR) repair) are perturbed [5]. It is important to note that DNA damage repair competence varies among individual breast tumors and closely correlates with chemosensitivity. For example, secondary mutations of BRCA1 or BRCA2 (essential factors in the HR pathway) caused by chemotherapy using cisplatin or poly(ADP-ribose) polymerase inhibitor in BRCA1/2-mutated cancers restore the wild-type reading frame and, therefore, the tumor acquires resistance to these drugs [6-8]. These facts indicate that chemosensitivity of BRCA-associated cancers could be strongly affected by DNA damage repair capability. Based on this evidence it has been suggested that HR competence could be a potential biomarker for chemosensitivity [9]. Rad51, a protein that plays a direct role in HR, especially reflects the HR competence of cells. Therefore, knowing its status is likely to be valuable when assessing HR competence in tumor cells in order to instruct therapeutic decisions [9].

The HR pathway for DSB repair is executed by sequential recruitment of repair proteins to chromatin around DNA lesions. Accumulation of the proteins is regulated by complex mechanisms that utilize phosphorylation and ubiquitination modifications mediated by kinases including ataxia telangiectasia mutated (ATM), and at least four ubiquitin E3 ligases, RNF8, RNF168, Rad18, and BRCA1 [10-17]. The Mre11-Rad50-Nbs1 complex first recognizes DSBs and recruits ATM. ATM then phosphorylates the histone variant H2AX (γH2AX) [18,19] that triggers accumulation of the downstream E3 ligases RNF8 [11-13,20] and RNF168 [14,15]. Lysine 63-linked polyubiquitin chains built at the sites of DNA damage by these E3 ligases next recruits the BRCA1-Abraxas-RAP80 complex through the RAP80 component, a protein that contains ubiquitin interacting motif domains [21-23]. BRCA1 is then essential in order to recruit repair effector proteins, including Rad51, that perform HR through sister chromatid exchange [24,25]. Depletion of any one of these proteins results in HR deficiency accompanied by loss of Rad51 focus formation, causing cells to become hypersensitive to DSB-inducing agents.

In this study we attempt to clarify the value of HR competence for the prediction of breast cancer chemosensitivity. One contention is that nuclear focus formation of repair proteins in baseline breast cancer tissues is a response to spontaneous DNA damage during cell proliferation and, in turn, may represent a marker of HR competence of cells to exogenous DNA damage. Therefore, it may predict tumor response to DNA damage-inducing chemotherapy such as with EC. Also, the focus formation after chemotherapy could provide us with additional information regarding the DNA damage-response capacity. To verify *in vivo *whether focus formation of repair proteins actually occurs in response to DNA damage-inducing chemotherapy and whether it correlates with tumor fates after chemotherapy, we analyzed foci in core needle biopsy specimens from breast cancer before and after neoadjuvant EC treatment.

## Materials and methods

### Patients and tumors

Sixty patients with primary breast cancer (2 cm or larger) who consecutively underwent neoadjuvant chemotherapy with EC followed by treatment with docetaxel (DOC) at the Division of Breast and Endocrine Surgery, St. Marianna University School of Medicine, Japan, were enrolled in the present study from August 2005 to July 2007. Tumor specimens were obtained by core needle biopsy prior to starting therapy and 18 to 24 hours after the first cycle of EC treatment. Informed consent for the additional core needle biopsy and experimental use of tumor samples was obtained for all patients in accordance with an approved Institutional Review Board application (registration number 946).

The chemotherapy regimen consisted of four 21-day cycles of EC (E: 80 mg/m^2 ^on day 1, C: 600 mg/m^2 ^on day 1) followed by four 21-day cycles of DOC (75 mg/m^2 ^on day 1). 75 mg/m^2 ^DOC was administrated four times as total (only on day 1). There was no increase or decrease of the dose. Tumor size was evaluated by three-dimensional images obtained by helical computed tomography CT scan with a teleradiologic image workstation (ZIOSTATION^®^, Ziosoft Inc., Tokyo, Japan) at baseline, 14 to 21 days after the last cycle of EC, and 21 days after the last cycle of DOC treatment. The effect of chemotherapy on the tumor was assessed as the three-dimensional volume reduction rate or tumor response rate. The tumor response was evaluated either by Response Evaluation Criteria in Solid Tumors (RECIST) [26] or by the three-dimensional volume evaluation defined as: complete response (CR; disappearance of the disease), partial response (PR; reduction of tumor volume of ≥65%), stable disease (SD; volume reduction <65% or enlargement ≤73%), or progressive disease (PD; volume enlargement ≥73%). These are equivalent to CR (disappearance), PR (reduction of ≥30%), SD (reduction <30% or enlargement ≤20%), or PD (enlargement ≥20%) in unidimensional RECIST criteria, respectively (reviewed in [27]). We also analyzed responses with a 50% border between PR and SD (instead of 65%) to evaluate more resistant cases.

### Immunohistochemical analysis

Immunohistochemical analysis was performed by the DAKO EnVision system (DAKO, Copenhagen, Denmark) with modifications. Formalin-fixed, paraffin-embedded specimens were cut and heated in a water bath (95°C, 40 minutes) in Target Retrieval Solution (pH 9.0, Dako, Carpinteria, CA, USA) for detection of BRCA-1 or in 10 mM sodium citrate buffer (pH 6.0) for γH2AX and Rad51. No pre-treatment was necessary to detect conjugated ubiquitin. After quenching of endogenous peroxidase, the sections were incubated overnight at 4°C with primary antibody at the appropriate dilution [Additional file 1], washed with PBS, and incubated with horseradish peroxidase-labeled polymer conjugated secondary antibody (EnVision+ System, Dako, Carpinteria, CA, USA) for 30 minutes at room temperature. Color development was achieved by 3, 3'-diaminobenzidine tetrahydrochloride. Effectiveness and specificity of each antibody for the detection of DNA damage-induced nuclear foci were verified with cultured cells treated with ionizing radiation (IR) or epirubicin. The immunofluorescent study has been previously described [28,29]. The nuclear foci were further analyzed with the protocol used in the tissue stain. The intrinsic subtype[30] was approximated by receptor status determined by standard immunohistochemical and fluorescence in situ hybridization (FISH) analyses: luminal A: ER+ and/or PR+ and HER2-; luminal B: ER+ and/or PR+ and HER2+; HER2: ER- and PR- and HER2+; triple negative: ER- and PR- and HER2-. Tumors that were immunochistochemically scored as 3+, or 2+ with FISH-positive, were regarded as positive for HER2 status. Cytokeratin (CK) 5/6 expression was also examined to evaluate the basal-like character.

### Immunohistochemical scoring

Taking into consideration that all immunohistochemical markers used in the study localize to sites of DNA damage in the normal HR pathway, we only counted cells displaying nuclear focus formation and disregarded cytoplasmic or diffuse nuclear staining. We scored the nuclear foci staining as follows: 0 = no positive cells, 1 = less than 10% positive cells, 2 = 10% or greater, but less than 80% positive cells, 3 = 80% or greater positive cells. Two observers (HA and HK) were blinded to the clinical information to avoid observer subjectivity when evaluating the immunohistochemical staining. To correlate staining with tumor response, we divided the cases into negative and positive samples to simplify the statistical analyses. The positive cases are a total of the categories with a foci score of 1, 2 and 3. To assess the capacity of the DNA damage response (DDR) using a more comprehensive approach, we configured the DDR score by counting the total number of positive factors present in baseline foci of BRCA1, γH2AX and Rad51, and EC-induced foci of Rad51, per case.

### Statistical analysis

The variables measured in the study were first investigated for association by the chi-squared contingency table analysis. For rank correlation, Spearman's method was performed to determine the correlation between the foci score of two repair proteins and to determine the correlation between tumor response rate and focus formation of each repair protein or DDR score. For parametric analyses of tumor volume reduction, Student's unpaired t-test and the Tukey-Kramer method were performed for two-factor comparisons and multiple comparisons, respectively. For evaluation of significance of DDR score and other clinicopathological factors in correlation with mean tumor volume reduction or tumor response rate, variant analysis (univariate) or logistic regression analyses (univariate and multivaliate), respectively, were performed. All analyses were carried out using Statview 5 statistical software (SAS Institute Inc, Cary, NC, USA). Statistical significance was declared for *P *values less than 0.05.

## Results

### Clinical and pathologic features

Sixty patients with primary breast cancer were included in the present series. All tumors were diagnosed as invasive ductal carcinoma. Patient clinical characteristics are given in Table 1. All triple-negative tumors were positive for CK5/6 (therefore described as basal-like in Table 1) whereas three cases of Luminal A, one case of Luminal B and three cases of HER2 type were positive for CK5/6. Three patients have one first-degree relative with a history of breast cancer and two patients have one second-degree relative with a history of breast or ovarian cancer. All patients completed an EC plus DOC regimen. Rad51 and γH2AX stains were not performed on tumor specimens before EC in two patients because of insufficient tumor sample after reserving stocks for clinical use. Tumor size evaluation by CT after treatment with EC plus DOC was not performed for one patient because of the patient's condition. All but one patient received breast surgery after EC and DOC.

**Table 1:** Patient characteristics

Characteristic factor	Number of patients	Characteristic factor	Number of patients
Age at treatment start		Cancer stage	
Median	50	I	0
Range	34-68	II	53
Lymph node metastasis		III	5
Negative	36	IV	2
Positive	24	Intrinsic subtype*	
Tumor stage		Luminal A	37
T1	0	Luminal B	6
T2	54	HER2	11
T3	6	Basal-like	6
T4	0	Total	60

* intrinsic subtypes were approximated by immunohistochemical receptor status. HER, human epidermal growth factor receptor.

### Nuclear foci staining of DNA damage repair proteins

**Figure 1:**
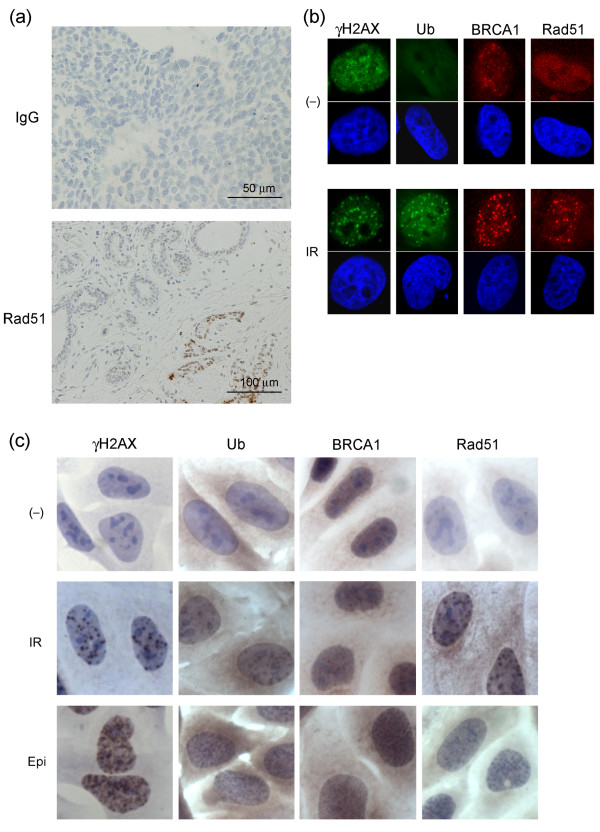
**Immunohistochemistry controls and antibody specificity**. **(a) **Immunohistochemical staining with control IgG for tumors after the first cycle of epirubicin plus cyclophosphamide (EC) treatment (upper panel). Lower panel shows Rad51 staining for morphologically diagnosed non-cancerous breast tissues (left upper part) and tumor (right lower part) after the first cycle of EC treatment. Although non-cancerous breast cells also expressed nuclear foci formation the number and intensity was significantly lower than that in tumor cells. **(b and c) **DNA damage-induced nuclear foci formations detected by antibodies used in the study. HeLa cells were either untreated (-), treated with 5 Gy ionizing radiation (IR) or 0.2 μg/ml epirubicin (Epi), incubated for three hours and fixed. Cells were then subjected either to immunofluorescent analyses with the indicated primary antibodies and **(b) **FITC- (green) or Rhodamine- (red) conjugated secondary antibodies, or **(c) **to the same protocol as that used in the tissue stain. For immunofluorescent analyses the nucleus was counterstained with DAPI. Ub, conjugated ubiquitin.

To assess the competence of the DSB repair pathway, we immunohistochemically analyzed γH2AX, conjugated ubiquitin, BRCA1, and Rad51 in nuclear foci based on the idea that these candidates may represent a typical course of the DSB repair cascade [31]. Of these, γH2AX is the most upstream element, sequentially followed in the cascade by conjugated ubiquitin, BRCA1, and Rad51. Rad51 is the most downstream of these four proteins and is directly involved in HR. However, it should be mentioned that DNA repair failure due to genes at the same level of or downstream of RAD51, such as RAD51AP1 [32] or translesion DNA polymerases [33,34], is an unlikely cause of loss of foci formation of these proteins. In addition to untreated, baseline breast cancer tissues, we analyzed the tissues 18 to 24 hours after the first cycle of EC treatment to obtain further information for the assessment of DNA repair capacity. The antibodies used in this study are commonly used and well characterized in general. In addition we tested background staining and confirmed the specific detection of nuclear foci at DSBs caused by IR or epirubucin treatment (Figure 1).

**Figure 2:**
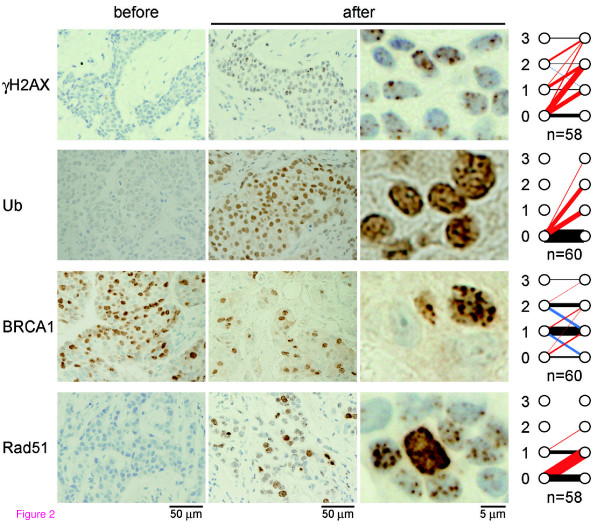
**Nuclear focus formation in response to chemotherapy**. Tumor specimens were obtained by core needle biopsy before and 18 to 24 hours after the first cycle of epirubicin plus cyclophosphamide (EC) treatment. Immunohistochemical findings from representative cases for γH2AX, conjugated ubiquitin (Ub), BRCA1, and Rad51 are shown. Graphs at right demonstrate changes in nuclear foci score after EC treatment in all cases analyzed. The red, blue, and black lines indicate cases with increased, decreased, and unchanged scores, respectively. The thickness of the lines proportionally reflects the number of cases. The thinnest line (γH2AX score 3 to 3) and the thickest line (Ub, score 0 to 0) represent 1 and 34 cases, respectively. n, number of cases analyzed.

The immunohistochemical analyzes revealed that in all but two cases, the foci score of at least one of the repair proteins was altered in response to EC treatment. Representative data for immunohistochemical findings of the nuclear focus formation of the repair proteins before and after the first cycle of EC are shown in Figure 2 with panels summarizing the foci scores of the cases. Prior to EC treatment, samples were stained to determine baseline staining of foci. The foci were positive for γH2AX (20 of 58 cases), BRCA1 (51 of 60 cases), or Rad51 (11 of 58 cases) whereas no cases exhibited foci staining for conjugated ubiquitin (0 of 60 cases). In response to EC treatment, the number of foci staining positive for γH2AX (44 of 58 cases), conjugated ubiquitin (26 of 60 cases), and Rad51 (31 of 58 cases) increased, whereas foci staining for BRCA1 either increased (9 of 60 cases), remained unchanged (38 of 60 cases) or decreased (13 of 60 cases). The reason why BRCA1 foci staining decreased after treatment in some cases is not clear at present but it could be implicated in the presence of BRCA1 foci in normal S-phase that colocalizes with proliferating cell nuclear antigen (PCNA) at DNA replication fork [35]. The foci score of BRCA1 after EC (EC-induced foci score) significantly correlated with that of Rad51 (*P *= 0.0017; Table 2), likely reflecting the requirement of BRCA1 for Rad51 recruitment at the site of DNA damage. However, no other correlations between repair proteins were observed, and no clear pattern combinations of repair proteins emerged.

**Table 2:** Correlation between EC-induced foci of Rad51 and BRCA1

	BRCA1				
	
	0	1	2	3	Total
Rad51					
0	7	10	2	0	19
1	3	24	9	2	38
2	0	0	2	0	2
Total	10	34	13	2	59
					*P *= 0.0017

*P *value is from the Spearman's rank correlation test. EC, epirubicin plus cyclophosphamide.

### Association of focus formation of each repair protein with tumor response to chemotherapy

To elucidate the possible association between DDR competence and tumor response to chemotherapy, we correlated the presence of individual repair proteins in foci with tumor volume before and after chemotherapy. Tumor volume was measured prior to chemotherapy to establish the baseline volume. The mean volume reduction of tumors after EC and after EC and DOC was 59.7 ± 25.8% and 76.0 ± 20.7% of baseline tumor volume, respectively. We analyzed the presence of repair proteins in foci before (baseline foci) and after EC treatment (EC-induced foci), sorted them into positive and negative foci groups for each individual repair protein, and then correlated each group with tumor volume [Additional file 2]. There was a significant difference in tumor volume after EC between BRCA1-positive and BRCA1-negative baseline foci groups (82.1 ± 17.8% vs 55.7 ± 25.1%, *P *= 0.0039) [Additional file 2a]. We then performed the same analysis after EC and DOC treatment. In addition to BRCA1 (93.7 ± 6.6% vs 72.8 ± 20.7%, *P *= 0.0044), significant differences in tumor volume were observed between positive and negative γH2AX (78.4 ± 17.4% vs 65.6 ± 26.8%, *P *= 0.0429) and Rad51 baseline foci groups (78.1 ± 18.9% vs 63.6 ± 24.4%, *P *= 0.0351) [Additional file 2b].

We next tested the correlation between scored foci groups and the tumor response rate. The tumor response rate was evaluated with RECIST or three-dimensional volume reduction using either 65% or 50% of the PR/SD border (as described in the Materials and Methods). Tumor responses to EC and EC plus DOC according to focus formation status are shown in Additional file 3. Contingency table analyses demonstrate significant differences in the EC tumor response rate between BRCA1-positive and BRCA1-negative baseline foci groups and between Rad51-positive and Rad51-negative EC-induced foci groups for all three criteria of the response rate (ZIO 65%, ZIO 50%, RECIST). There continued to be a significant difference in tumor response rate after EC and DOC treatment between Rad51-positive and Rad51-negative EC-induced foci groups for all three criteria. In addition, when evaluated with a three-dimensional volume reduction using 50% of the PR/SD border, significant differences in the tumor response rate to EC and DOC were observed between Rad51-positive and Rad51-negative baseline foci groups.

To specify the correlation of these focus formation groups with tumor response rates, we further analyzed the data with Spearman's rank correlation method. When evaluated with three-dimensional volume reduction using 50% of the PR/SD border, Spearman's analysis showed that the presence of BRCA1-positive baseline foci associated with poor EC tumor response (*P *= 0.0067) [Additional file 3]. Spearman's analysis also demonstrated that the presence of Rad51-positive baseline foci (*P *= 0.0078) or EC-induced foci (*P *= 0.0042) [Additional file 3] associated with poor EC and DOC tumor response.

### Association of DDR score with tumor reduction by chemotherapy

**Figure 3:**
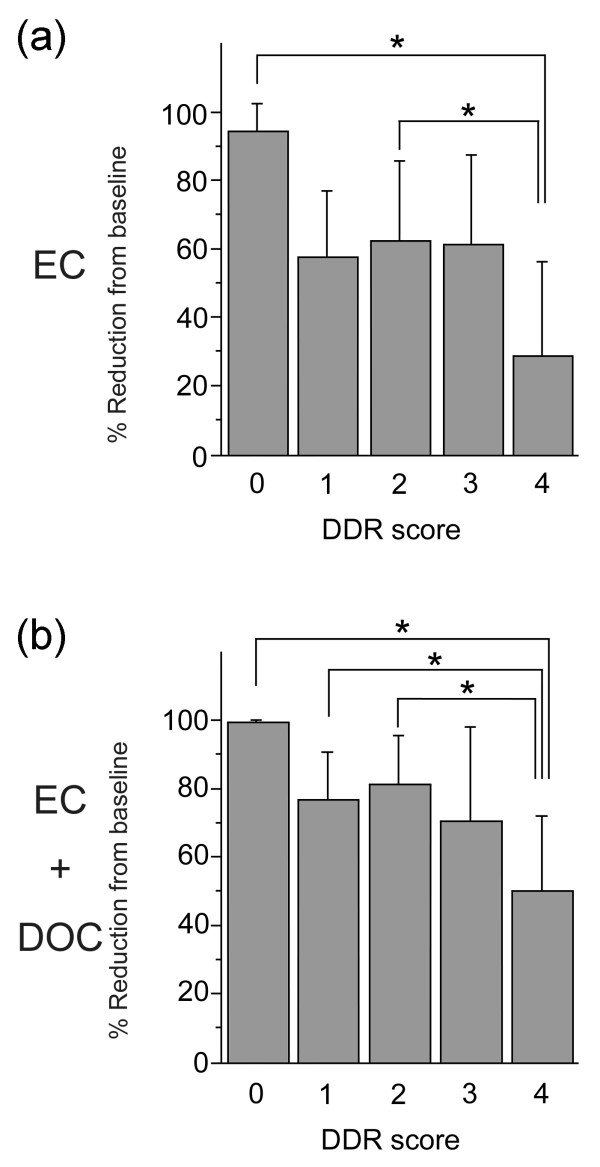
**Mean tumor volume reductions after (a) EC or (b) EC and DOC according to DDR score**. Error bars represent standard deviation. Significance was analyzed by Tukey-Kramer test setting *P *< 0.05 as significance threshold. DDR, DNA damage response; DOC, docetaxel; EC, epirubicin plus cyclophosphamide.

The analysis correlating focus formation of BRCA1, γH2AX, and Rad51 prior to treatment and of Rad51 foci after EC treatment with the mean tumor volume reduction or tumor response rate [Additional files 2 and 3] uncovers a significant inverse correlation with tumor response for each of the four conditions. These data support the supposition that higher DDR competency produces tumors resistant to chemotherapy. To correlate overall DDR competency with tumor reduction, we devised a simple measurement to assess DDR competency. Each patient case was analyzed for the presence of all four of the above listed conditions and was assigned a DDR score of 0 to 4 based on the number of conditions present. This DDR score was then correlated with mean tumor volume reductions. Number of cases in each DDR score is shown in Table 3. As shown in Figure 3, both the mean tumor volume reductions after EC (28.4 ± 28.1%) and after EC and DOC (49.9 ± 22.0%) for DDR score 4 (all four conditions present) were the lowest among all the scores. There were significant differences between score 4 and either score 0 or 2 for the mean tumor volume reductions after EC (Figure 3a) and between score 4 and either score 0, 1 or 2 for the mean tumor volume reductions after EC and DOC (Figure 3b), as judged by the Tukey-Kramer multiple comparisons study setting *P *< 0.05 as a significance threshold. In addition, Spearman's analysis showed that a high DDR score was associated with poor tumor response rate after EC and DOC (*P *= 0.0031) when evaluated with three-dimensional volume reduction using 50% of the PR/SD border (Table 3). A high DDR score also tended to be associated with poor tumor response rate after EC (*P *= 0.0639, Table 3).

**Table 3:** Correlation between DDR score and tumor response rate (ZIO 50%)

	DDR score					
	
	0	1	2	3	4	Total
After EC						
CR	1	0	1	0	0	2
PR	1	12	14	9	2	38
SD	0	3	8	3	4	18
Total	2	15	23	12	6	58
						*P *= 0.0639
After EC + DOC						
CR	1	1	3	1	0	6
PR	1	13	19	9	2	44
SD	0	0	1	2	4	7
Total	2	14	23	12	6	57
						*P *= 0.0031

*P *values are from the Spearman's rank correlation test. CR, complete response; DDR, DNA damage response; EC, epirubicin plus cyclophosphamide; PR, partial response; SD, stable disease.

The correlation between DDR score and the tumor response prompted us to examine whether it has a significant impact among other clinicopathological factors including age, cancer stage, tumor size, nodal metastatic status, and subtypes. None of these factors correlated with DDR score (data not shown). The variant analysis for mean tumor volume reduction after EC revealed that only DDR score (*P *= 0.0069), but no other factors correlated with the mean tumor volume reduction (Table 4). The variant analysis for mean tumor volume reduction after EC and DOC also demonstrated DDR score (*P *= 0.0035) as the most significant correlation factor, followed by nodal status (*P *= 0.0201) and tumor size (*P *= 0.0538, Table 4). In addition, univariate logistic regression analysis showed that a high (3 and 4) DDR score was most significantly associated with poor tumor response after EC and DOC (*P *= 0.0095) when evaluated with volume reduction using 50% of the PR/SD border, followed by tumor size (*P *= 0.0260), cancer stage (*P *= 0.0465), and subtype (*P *= 0.0659, Table 5). We then examined multivariate analysis with tumor size, nodal status, subtype, and DDR score, the factors that showed probable association with tumor response rate in the univariate analysis. Cancer stage was omitted because it was correlated with tumor size. Importantly the result indicated that only the DDR score was significantly associated with tumor response rate (*P *= 0.0402) independent of other factors analyzed (Table 6).

**Table 4:** Univariate analysis of variance for mean tumor volume reduction

		*n*	Mean tumor volume reduction (%) ± SD	*P*
After EC				
	Age (years)			
	-50	30	64.1 ± 25.9	*0.1710*
	51-	29	54.9 ± 25.4	
	Cancer stage			
	II	53	61.5 ± 25.1	*0.0962*
	III	5	55.7 ± 29.1	
	IV	2	21.9 ± 6.4	
	Tumor stage			
	T2	54	60.8 ± 25.3	*0.3059*
	T3	6	21.9 ± 6.4	
	Nodal status			
	N-	24	62.7 ± 24.9	*0.4557*
	N+	36	57.6 ± 26.6	
	Subtype			
	Luminal A	37	58.8 ± 21.3	*0.2923*
	Luminal B	6	72.9 ± 9.4	
	HER2	11	50.4 ± 36.4	
	Basal-like	6	69.4 ± 37.0	
	DDR score			
	0	2	94.6 ± 7.6	*0.0069*
	1	15	57.3 ± 19.4	
	2	23	62.8 ± 22.9	
	3	12	61.1 ± 26.6	
	4	6	28.4 ± 28.1	
After EC-DOC				
	Age			
	-50	30	80.6 ± 23.9	*0.0804*
	51-	29	71.2 ± 15.7	
	Cancer stage			
	II	52	77.9 ± 19.8	*0.1230*
	III	5	64.3 ± 25.7	
	IV	2	54.7 ± 17.6	
	Tumor stage			
	T2	53	77.7 ± 19.7	*0.0538*
	T3	6	60.6 ± 24.7	
	Nodal status			
	N-	23	83.7 ± 14.3	*0.0201*
	N+	36	71.0 ± 22.7	
	Subtype			
	Luminal A	36	77.9 ± 16.1	*0.0789*
	Luminal B	6	86.0 ± 9.5	
	HER2	11	62.4 ± 30.5	
	Basal-like	6	79.7 ± 24.8	
	DDR score			
	0	2	99.6 ± 0.6	*0.0035*
	1	14	77.2 ± 13.2	
	2	23	81.0 ± 14.7	
	3	12	70.7 ± 27.5	
	4	6	49.9 ± 22.0	

DDR, DNA Damage Response; DOC, docetaxel; EC, epirubicin plus cyclophosphamide; HER, human epidermal growth factor receptor; SD, standard deviation.

**Table 5:** Univariate logistic regression analysis of factors affecting tumor response rate (ZIO 50%)

		Odds ratio	(95% CI)	*P*
After EC + DOC				
	Age (years)			
	<51	1.000		
	51≤	0.800	(0.192-3.333)	*0.7592*
	Cancer stage			
	II	1.000		
	III, IV	5.750	(1.028-32.174)	*0.0465*
	Tumor stage			
	T2	1.000		
	T3	7.833	(1.279-47.964)	*0.0260*
	Nodal status			
	N-	1.000		
	N+	6.286	(0.730-54.110)	*0.0942*
	Subtype			
	Luminal A, B	1.000		
	HER2, Basal-like	3.958	(0.913-17.154)	*0.0659*
	DDR score			
	0, 1, 2	1.000		
	3, 4	9.423	(1.729-51.359)	*0.0095*

CI, confidence interval; DDR, DNA Damage Response; DOC, docetaxel; EC, epirubicin plus cyclophosphamide; HER, human epidermal growth factor receptor.

**Table 6:** Multivariate logistic regression analysis of factors affecting tumor response rate (ZIO 50%)

		Odds ratio	(95% CI)	*P*
After EC + DOC				
	Tumor stage			
	T2	1.000		
	T3	2.246	(0.290-17.420)	*0.4388*
	Nodal status			
	N-	1.000		
	N+	3.651	(0.346-38.506)	*0.2813*
	Subtype			
	Luminal A, B	1.000		
	HER2, Basal-like	2.484	(0.464-13.287)	*0.2874*
	DDR score			
	0, 1, 2	1.000		
	3, 4	6.694	(1.088-41.182)	*0.0402*

CI, confidence interval; DDR, DNA Damage Response; DOC, docetaxel; EC, epirubicin plus cyclophosphamide; HER, human epidermal growth factor receptor.

## Discussion

In the present study using human tumor specimens we show for the first time that DNA repair competence may predict breast cancer sensitivity to DNA damage-inducing chemotherapy. We selected γH2AX, conjugated ubiquitin, BRCA1, and Rad51, proteins in the DSB repair cascade, to assess DNA repair competence because accumulated evidence demonstrates that inactivation of genes in the DSB repair pathway results in cellular sensitivity to DNA damage-inducing chemotherapy [16,29,31,36-38]. In our study, these repair proteins dramatically responded to EC treatment. The conjugated ubiquitin response was especially dramatic as approximately half of the cases analyzed formed conjugated ubiquitin foci, compared with undetectable foci formation prior to treatment. This suggests that ubiquitination occurs *in vivo *during the DNA damage response in an early stage after chemotherapy. However, in spite of the dramatic response, we did not find any significant correlation between conjugated ubiquitin foci formation and tumor response. The reason is currently unknown. One possibility is that this could be attributed to the fact that ubiquitination is also involved in DNA damage response pathways other than for DSBs.

We did not find certain trends of the combinations of responding repair proteins. Several reasons could account for this observation. First, the metabolism and pharmacokinetics of the agents could vary per patient. The ideal time to obtain the *in vivo *sample was, therefore, difficult to determine. The experimental design employed in this study was not very robust in this way. In cultured cells, γH2AX accumulates at sites of DNA damage just minutes after the damage occurs, whereas BRCA1 and Rad51 foci appear 30 minutes to several hours afterwards [11,35,39,40]. In this study we harvested samples 18 to 24 hours after EC treatment because the agents were still expected to be present in patients and we also considered the patient's convenience. However, the ideal timing remains to be determined if biopsy after chemotherapy is required.

The second reason for the diversity of the DDR response could be attributed to the diversity of aberrations of the genes responsible for DSB repair in each breast cancer. Theoretically, defects in the recruitment of upstream repair proteins could result in loss of downstream proteins at sites of DNA damage, and this has been shown to be the case in many molecular biological studies using cultured cells [10-15,21-23]. Furthermore, it was also shown that Rad51 nuclear expression is absent in tumors associated with *BRCA2 *mutation [41]. The positive correlation found between EC-induced BRCA1 and Rad51 foci in this study (Table 2) may also support this interpretation. In contrast, it was reported that overexpression of Rad51 restored Rad51 focus formation and rescued the sensitivity of *BRCA1*-deficient cells to x-rays and cisplatin [42]. Importantly, up-regulation of Rad51 was a common feature of *BRCA1*-deficient breast tumors [42]. These data suggest that the mechanism of DSB repair response *in vivo *is not simple and that assessment of DSB repair aberrations in each patient case is, therefore, unreasonable at present.

In an attempt to address this problem in our current study, we assessed the comprehensive capacity of DSB repair by incorporating multiple candidate factors into one DDR score. We found that foci of BRCA1, γH2AX, and Rad51 prior to treatment and EC-induced foci of Rad51 correlated with tumor response when compared either with the mean tumor volume reduction or the tumor response rate. When incorporating these four factors into one DDR score a significant correlation was observed with mean tumor volume reduction after EC, whereas no other factors correlated with the mean tumor volume reduction (Table 4 and Figure 3a). Although it was not statistically significant, the similar correlation was also observed between DDR score and tumor response rate (Table 3). These correlations became more significant after EC and DOC treatment (Tables 3 to 5 and Figure 3b) and the DDR score was an independent predictive factor of other factors including tumor subtype when evaluated with volume reduction using 50% of the PR/SD border (Table 6). Recent studies suggested that luminal tumors have low response rate to neoadjuvant chemotherapy, whereas basal-like and HER2+ tumors have higher response rates. For example, it has been reported that clinical response rate (CR and PR) to anthracyclin-based chemotherapy of luminal A was 39%, whereas that of basal-like, which has been implicated with BRCA1 dysfunction [43,44], was 85% [45]. The response rates to EC treatment of luminal A (15 of 37 cases, 40.5%) and basal-like (4 of 6 cases, 66.7%) subtypes in the current study were not very different from the previous report. However, we could not find any correlation between subtype and DDR score while DDR score independently predicted the chemosensitivity. The result may reflect the fact that luminal A tumors also include DNA damage-sensitive tumors with defective HR pathways that can be counted by the DDR score. Supporting this it has been shown that tumors caused by BRCA2 deficiency mainly become luminal A tumors [44,46,47].

The reason why the correlation between the DDR score and tumor response after EC and DOC treatment became more significant than that after EC is not clear at present. As DOC does not induce DNA DSBs, the observed effect is not likely to be due to the sensitivity to DNA damage in those tumors. DOC might be more toxic for the cells with gross genomic aberration caused by the pretreatment with EC under the condition of being less HR competent. Alternatively it is possible that time length after EC treatment enhanced the difference of the outcome.

Interestingly, DDR score group 4 consisted of cases with poor tumor responses to chemotherapy when evaluated for both mean tumor volume reduction (Figure 3) and tumor response rate (Table 3). This result may lead to the possibility of using DDR status in the clinic to predict and exclude non-responders to EC treatment. It is noteworthy to point out that the HR repair cascade for DSB contains many essential proteins other than those tested in this study. By including select subsets of proteins for analysis, it may be possible to identify non-responders in order to avoid unnecessary chemotherapy. Ideally in such cases, the levels of baseline foci present prior to treatment would provide enough information to determine appropriate treatment, preventing the need for additional core needle biopsy after chemotherapy.

## Conclusions

In conclusion, our results suggest the importance of evaluating DDR competence to predict breast cancer chemosensitivity and warrant further investigation into its effectiveness as a way to exclude non-responding patients.

## Abbreviations

CK: cytokeratin; CR: complete response; CT: computed tomography; DDR: DNA damage response; DOC: docetaxel; DSB: DNA double-strand break; EC: epirubicin plus cyclophosphamide; ER: estrogen receptor; HER: human epidermal growth factor receptor; HR: homologous recombination repair; IR: ionizing radiation; PBS: phosphate-buffered saline; PD: progressive disease; PR: partial response; PR (+ or -): progesterone receptor (+ or -); RECIST: Response Evaluation Criteria in Solid Tumors; SD: stable disease.

## Competing interests

The authors declare that they have no competing interests.

## Authors' contributions

HA analyzed the majority of the data. HK conducted immunohistochemical analyses. AK obtained the data for tumor response. MT supported immunohistochemical analyses. WW characterized antibody specificities. HI and MF made substantial contributions to analysis and interpretation of the data. TO designed and conducted the studies, and wrote the manuscript. All authors read and approved the final manuscript.

## Supplementary Material

Additional file 1 **Table S1**. Antibodies used in the present immunohistochemical study. Click here for file

Additional file 2 **Figure S1**. Mean tumor volume reduction after EC (a) or EC+DOC (b) according to the nuclear foci status for DNA repair proteins. Click here for file

Additional file 3 **Table S2**. Tumor response rate according to the nuclear foci status for DNA repair proteins. Click here for file
