# Supplementary material for: Prediction of breast cancer sensitivity to neoadjuvant chemotherapy based on status of DNA damage repair proteins
Source: Breast Cancer Res. 2010 Mar 5;12(2):R17. doi: 10.1186/bcr2486 (PMC2879561; doi:10.1186/bcr2486)
Supplement: Additional file 1 — Table S1. Antibodies used in the present immunohistochemical study. [file bcr2486-S1.PDF]

Additional data file 1. Antibodies used in the present immunohistochemical study

Antibody	Clone	Dilution	Supplier
γH2AX	JBW301	1:200	Up State
Conjugated Ubiquitin	FK2	1:200	Nippon Bio-Test
BRCA1	MS110	1:70	Calbiochem
Rad51	Polyclonal rabbit	1:400	Santa Cruz
