# Supplementary material for: Prediction of breast cancer sensitivity to neoadjuvant chemotherapy based on status of DNA damage repair proteins
Source: Breast Cancer Res. 2010 Mar 5;12(2):R17. doi: 10.1186/bcr2486 (PMC2879561; doi:10.1186/bcr2486)
Supplement: Additional file 2 — Figure S1. Mean tumor volume reduction after EC (a) or EC+DOC (b) according to the nuclear foci status for DNA repair proteins. [file bcr2486-S2.PDF]

Additional data file 2

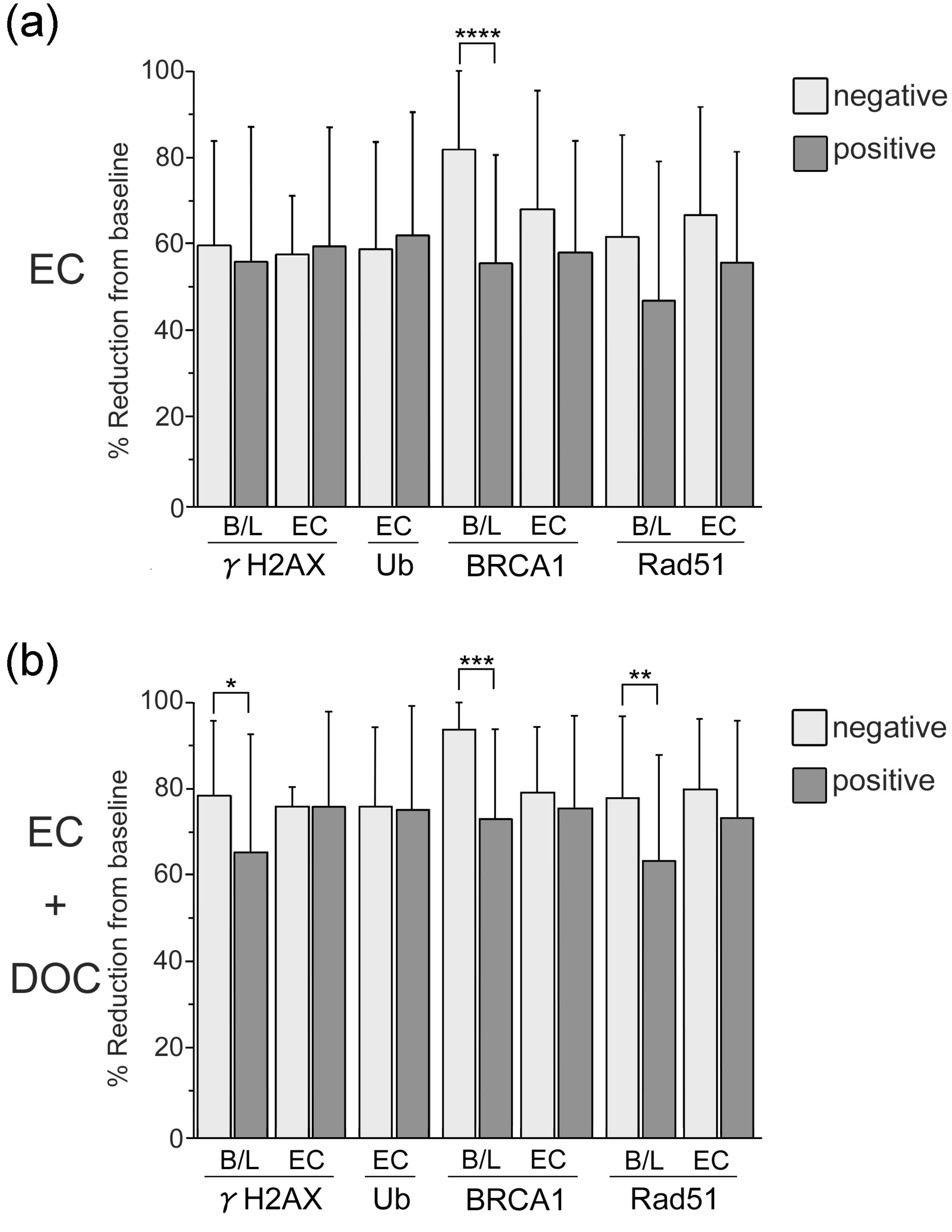

Additional data file 2. Mean tumor volume reduction after EC (a) or EC+DOC (b) according to the nuclear foci status for γH2AX, conjugated-ubiquitin (Ub), BRCA1, and Rad51. Dark bar, positive foci group; light bar, negative foci group (see Material and Methods for scoring criteria). Error bars represent standard deviation. Significance was analyzed by Student's t-test. *p=.0429, **p=.0351, ***p=.0044, ****p=.0039. Abbreviations: B/L, baseline foci; EC, EC-induced foci; n, number of cases.
