# Supplementary material for: Prediction of breast cancer sensitivity to neoadjuvant chemotherapy based on status of DNA damage repair proteins
Source: Breast Cancer Res. 2010 Mar 5;12(2):R17. doi: 10.1186/bcr2486 (PMC2879561; doi:10.1186/bcr2486)
Supplement: Additional file 3 — Table S2. Tumor response rate according to the nuclear foci status for DNA repair proteins. [file bcr2486-S3.PDF]

Additional data file 3. Tumor response rate according to focus formation status.

	γH2AX				Ub		BRCA1				Rad51			
	B/L		EC		EC		B/L		EC		B/L		EC	
	-	+	-	+	-	+	-	+	-	+	-	+	-	+
after EC														
(RECIST)														
CR	1	1	0	3	1	2	2	1	1	2	2	0	3	0
PR	17	7	2	23	15	10	4	21	5	20	20	4	6	18
SD	26	6	7	25	18	14	3	29	5	27	25	7	10	22
P	.4563		.2623		.6769		.0288		.7211		.6953		.0318	
(ZIO 65%)														
CR	1	1	0	3	1	2	2	1	1	2	2	0	3	0
PR	19	5	3	22	14	11	5	20	5	20	20	4	8	16
SD	24	8	6	26	19	13	2	30	5	27	25	7	8	24
P	.6419		.5890		.6821		.0130		.7211		.6953		.0289	
(ZIO 50%)														
CR	1	1	0	3	1	2	2	1	1	2	2	0	3	0
PR	30	8	7	32	20	19	7	32	8	31	32	6	12	26
SD	13	5	2	16	13	5	0	18	2	16	13	5	4	14
P	.5891		.6038		.2346		.0077		.5484		.4433		.0281	
* *P*	.8610		.7948		.0916		.0067		.2878		.2140		.0754	
after EC+DOC														
(RECIST)														
CR	5	1	0	7	3	4	2	5	1	6	6	0	5	2
PR	32	8	7	34	26	15	7	34	6	35	33	7	9	31
SD	6	5	2	9	4	7	0	11	3	8	7	4	4	7
P	.1977		.4873		.2095		.2181		.5992		.1674		.0327	
(ZIO 65%)														
CR	5	1	0	7	3	4	2	5	1	6	6	0	5	2
PR	30	8	9	30	24	15	7	32	7	32	32	6	9	29
SD	8	5	0	13	6	7	0	13	2	11	8	5	4	9
P	.4046		.0657		.4754		.1721		.9581		.0907		.0023	
(ZIO 50%)														
CR	5	1	0	7	3	4	2	5	1	6	6	0	5	2
PR	35	8	9	35	27	17	6	38	8	36	37	6	13	30
SD	3	5	0	8	3	5	1	7	1	7	3	5	0	8
P	.1354		.1917		.1888		.3290		.4145		.0166		.0014	
* *P*	.0766		.9999		.5935		.1405		.4800		.0078		.0042	

Abbreviations: B/L, baseline foci; EC, EC-induced foci.

P values are from the χ2 test unless otherwise indicated

*P values are from the Spearman's rank correlation test
